# Production of graphene oxide from pitch-based carbon fiber

**DOI:** 10.1038/srep11707

**Published:** 2015-07-09

**Authors:** Miyeon Lee, Jihoon Lee, Sung Young Park, Byunggak Min, Bongsoo Kim, Insik In

**Affiliations:** 1 Department of Chemistry, KAIST, Daejeon, 305-701, South Korea; 2 50 Daehak-ro, Department of Polymer Science and Engineering, Korea National University of Transportation, Chungju-si, Chungbuk, 380-702, South Korea; 3 50 Daehak-ro, Department of Chemical and Biological Engineering, Korea National University of transportation, Chungju-si, Chungbuk, 380-702, South Korea; 4 50 Daehak-ro, Department of IT Convergence (BK PLUS 21), Korea National University of Transportation, Chungju-si, Chungbuk, 380-702, South Korea

## Abstract

Pitch-based graphene oxide (p-GO) whose compositional/structural features are comparable to those of graphene oxide (GO) was firstly produced by chemical exfoliation of pitch-based carbon fiber rather than natural graphite. Incorporation of p-GO as nanofillers into poly(methyl methacrylate) (PMMA) as a matrix polymer resulted in excellent mechanical reinforcement. p-GO/PMMA nanocomposite (1 wt.-% p-GO) demonstrated 800% higher modulus of toughness of neat PMMA.

Graphene-based materials have received extensive research interests due to their superior electrical, thermal, mechanical, and optical properties based on their 2-dimensional (2D) planar structures having π−conjugation^1^. While bottom-up methods such as chemical vapor deposition have provided single- or few-layered graphene film with minimum defect sites^2^, top-down methods utilizing chemical^3^ or physical exfoliation^4^ of graphite are much beneficial for the mass production of quasi-2D carbon sheets which have recently attracted increasing attention as nanofillers for polymer nanocomposite. Until now, only natural or synthetic graphite has been successfully utilized for the mass production of 2D graphene sheets^5^. Therefore, it is quite interesting to evaluate the utilization of other sp^2^-carbon sources such as petroleum pitch-based carbon fiber (p-CF) for the formulation of graphene-like 2D carbon sheets. p-CF is prepared by sequential carbonization and graphitization of petroleum pitch on stretching and therefore p-CF has high carbon content more than 99%^6^. Scanning electron microscopy (SEM) image of acid-treated p-CF shown the presence of lots of stacked 2D carbon sheets along with the long axis of p-CF as shown in Fig. 1. Recent report by Peng *et al.*^7^ also confirms the presence of significant amounts of 2D conjugated *sp*^2^ domains in p-CF. Graphene quantum dots with lateral sizes between 1 and 5 nm were successfully obtained from p-CF, showing the size-dependent fluorescence of graphene quantum dots. From all these observations, p-CF can be regarded as an alternative *sp*^2^-carbon rich platform for the mass production of 2D graphene-like materials. In this work, chemical exfoliation of p-CF by typical modified Hummers method is attempted to provide p-GO as shown in Fig. 2. p-GO resembles the chemical and physical properties of GO which is typically obtained by the chemical exfoliation of natural graphite. Incorporation of p–GO as nanofillers into PMMA matrix produces p-GO/PMMA nanocomposite with dramatically enhanced modulus of toughness more than 800% even with 1 wt.-% inclusion of p-GO into the nanocomposite, demonstrating the effectiveness of p-GO as alternative 2D nanofiller comparable to GO.

## Results

### Preparation and chemical reduction of p-GO

**Figure 3:**
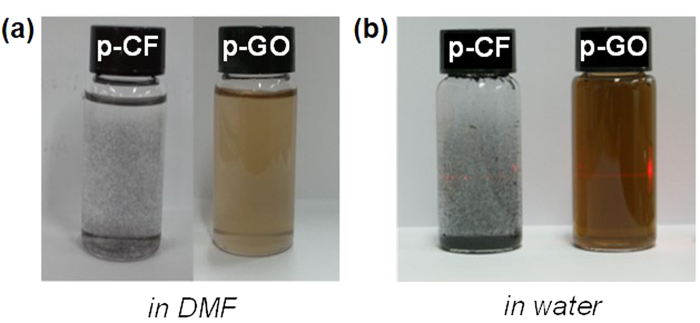
Photo images of p-CF and p-GO dispersions in (**a**) DMF (0.1 mg/L) and (**b**) water (1 mg/mL).

**Figure 4:**
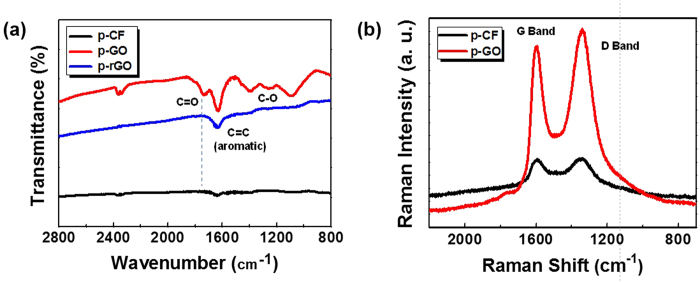
Photo images of p-CF and p-GO dispersions in (**a**) DMF (0.1 mg/L) and (**b**) water (1 mg/mL).

**Figure 5:**
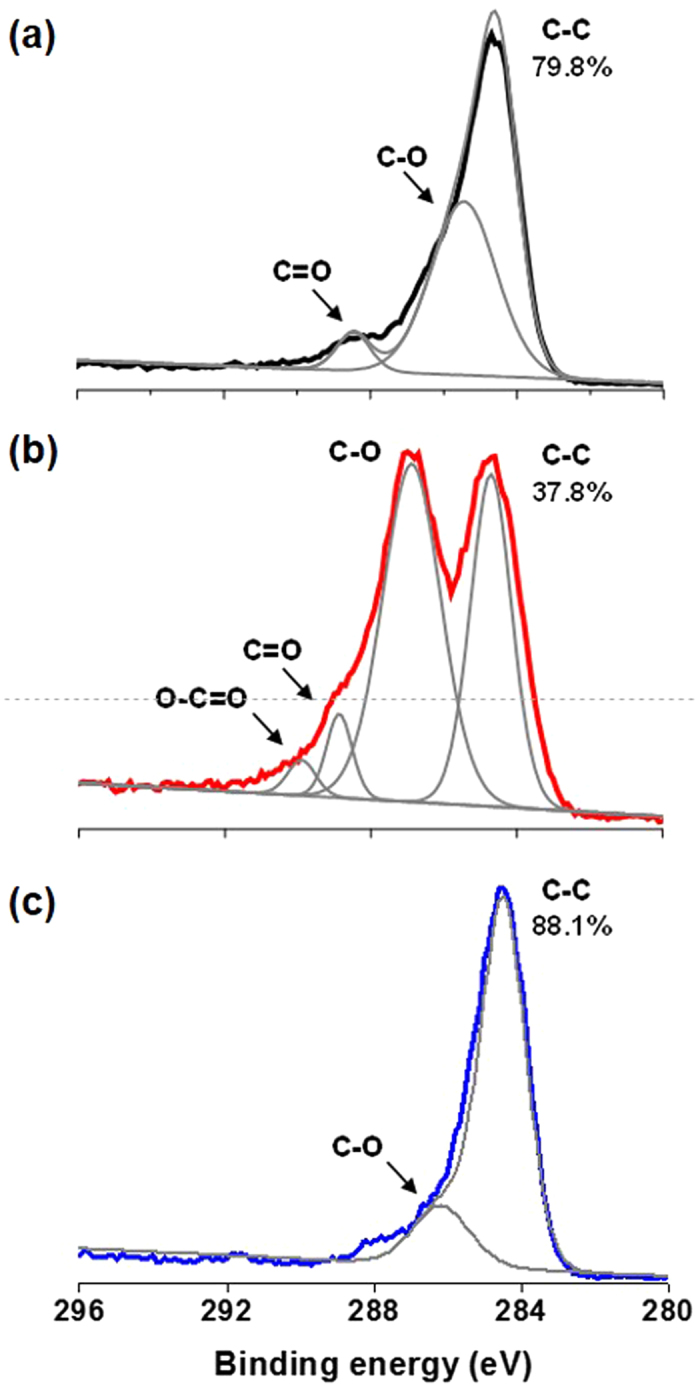
C1s XPS spectra of (**a**) p-CF, (**b**) p-GO, and (**c**) p-rGO (normalized with respect to the C-C peak).

**Figure 6:**
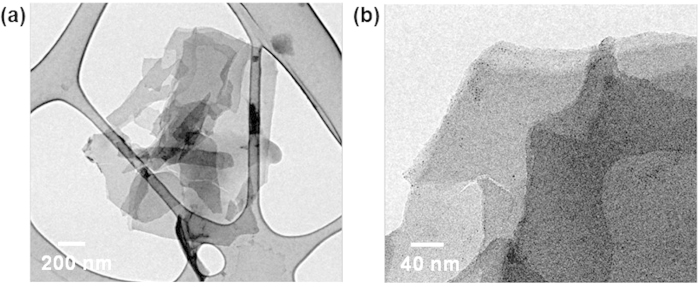
TEM images of (**a**) p-GO and (**b**) its magnification (on TEM grid with lacey carbon support films from Ted Pella, Inc.).

**Figure 7:**
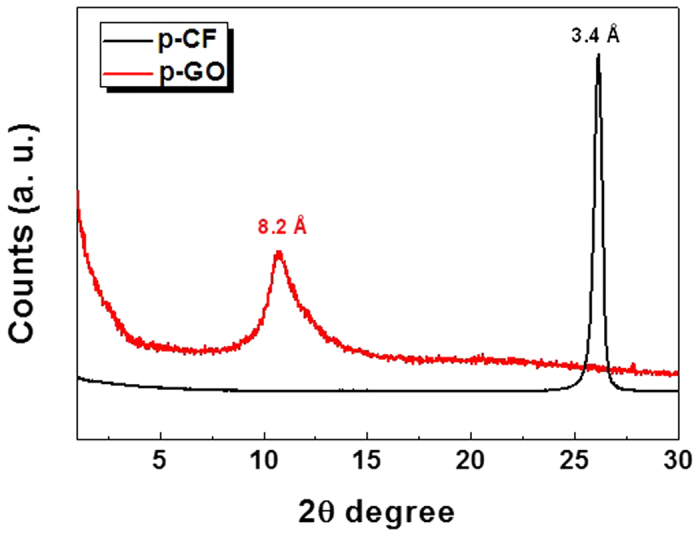
XRD spectra of p-CF and p-GO (1.54059 Å Cu Kα 1 as wavelength).

As GO can be prepared from chemical exfoliation of graphite, chemical exfoliation of p-CF (diameter of 10 μm and length up to 200 μm) was attempted through modified Hummers’ method^8^. At first, pre-treated p-CF was prepared through chemical oxidation of pristine p-CF by sulfuric acid and K_2_S_2_O_8_ in the presence of P_2_O_5_. Then, above pre-treated p-CF was further oxidized by both sulfuric acid and KMnO_4_, resulting in highly oxidized carbon material. Decomposing excess KMnO_4_ by washing with hydrochloric acid and deionized water produced dark brown solid material, p-GO. After drying overnight in vacuum, 7.5 g of p-GO is obtained from 5 g of p-CF. Compared with the production of GO which utilizes graphite as a source, it is noteworthy that there is significantly less under-oxidized material which is not soluble in water. Most of p-GO was completely dispersible in water and dimethyl formamide (DMF) while p-CF itself is not dispersible in any solvent media as shown in Fig. 3. To evaluate the compositional features of p-GO, both FT-IR and Raman spectra of p-GO were characterized. FT-IR spectrum of p-GO showed peaks for hydroxy stretching (O-H, 3429 cm^−1^), C=O stretching (carbonyl or carboxyl, 1734 cm^−1^), aromatic C=C bond stretching (1630 cm^−1^), carboxy C-O stretching (1397 cm^−1^), epoxy C-O stretching (1256 cm^−1^), and alkoxy C-O stretching (1096 cm^−1^) as shown in Fig. 4(a). The presence of significant amounts of polar functional groups (-OH, -COOH) supports hydrophobicity of prepared p-GO which is readily soluble both in water and DMF, revealing that typical Hummers method for the synthesis of graphite oxide or GO from natural graphite effectively operates for the preparation of p-GO from p-CF. The ratio of Raman D to G band intensity (ID/IG) increases from 1.01 for p-CF to 1.07 for p-GO as shown in Fig. 4(b), indicating a decrease in the average size of sp^2^ domains in p-GO compared with pristine p-CF. Because D band peak is relating with defects in graphene-based materials, the presence of significant D band peaks both in p-CF and p-GO suggests that p-CF itself has certain defect sites which are not present in Raman spectrum of graphite^9^. Meanwhile, presence of clear G band peaks both in p-CF and p-GO compromises that C=C backbone structure is maintained in them because G band peak is relating with C(sp^2^)-C(sp^2^) bond stretching vibrations^10^. Above compositional features of p-GO was confirmed by monitoring XPS spectra of both p-CF and p-GO. The degree of oxidation was calculated by deconvoluting C1s spectra into four peaks. By combining peak areas for C-O (epoxy or hydroxy, 286.2 eV), C=O (carbonyl, 287.8 eV), and O-C=O (carboxylate, 289.0 eV) functional groups, it is regarded that p-GO has 63.2% of oxidized carbon and 37.8% of graphitic carbon as shown in Fig. 5(b). The degree of oxidation for p-GO is slightly higher compared with that of GO (typically 50% of oxidized carbon) which is obtained by chemical exfoliation methods such as Hummers method, showing that p-GO has much enriched oxidized functional groups compared with GO^11^. Meanwhile, p-CF showed more than 79.8% of graphitic carbon as shown in Fig. 5(a), which shows the usefulness of p-CF as an alternative source material for the production of graphene-relating materials. The structural features of p-GO were examined by AFM, TEM, and XRD. AFM image of p-GO showed isolated 2D plates having the thickness of 1.2 nm as shown in Fig. S1 (see the Supporting information). Because typical hydrated GO shows thickness of 1.0 ~ 1.1 nm^12^, it is regarded that p-GO has comparable 2D planar to GO having single atomic carbon layer. Meanwhile, aspect ratio (relative length ratio of long axis to short axis) of each p-GO plate was found to be between 1.0 and 1.3 regardless of high aspect ratio of 20 in the case of pristine p-CF. TEM analysis of p-GO showed the presence of individual 2D sheets, up to 1 μm in diameter as shown in Fig. 6. Although p-GO plates in either above TEM and SEM images as shown in Fig. S2. are aggregated and crumpled, it is clearly sheet-like in images. Finally, XRD spectrum of p-GO supports that chemical exfoliation of p-CF into p-GO is successful. While pristine p-CF showed graphitic peak at 3.4 Å, p-GO showed broad scattering peak centered at 8.2 Å together with trace of graphitic peak at 3.4 Å as shown in Fig. 7. Therefore, it is evident that chemical exfoliation of p-CF through modified Hummers method effectively provides 2D planar p-GO which has very similar compositional and structural features comparable to GO^13^.

### Chemical reduction of p-GO

**Figure 8:**
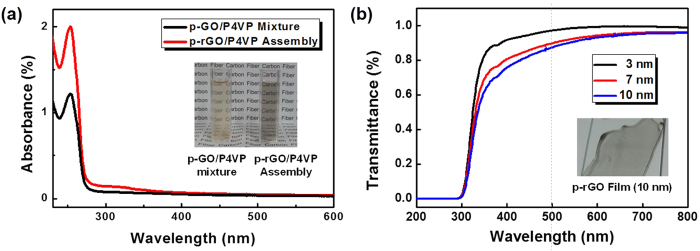
(**a**) UV-Vis spectra of both p-GO/P4VP mixture and p-rGO/P4VP assembly solutions, and (**b**) optical transmittance of p-rGO thin films.

The compositional and structural similarities between p-GO and GO prompt us to investigate the formulation of soluble chemically reduced p-GO (p-rGO) through noncovalent interaction with aliphatic polymer having aqueous solubility^14^,^15^,^16^. To accomplish this, aqueous p-GO solution was mixed with aqueous P4VP solution with weight ratio of 1:10 (p-GO:P4VP), forming p-GO/P4VP mixture solution^17^. Addition of excess hydrazine and subsequent heating at 80 °C for 24 hrs produced soluble p-rGO/P4VP assembly solution with dispersion stability more than 6 months. UV-Vis spectra of p-rGO/P4VP assembly solution showed increased optical absorption compared with p-GO/P4VP mixture solution as shown in Fig. 8(a). Interestingly, p-rGO showed λ_max_ at 254 nm, while p-GO showed λ_max_ in the 227–231 nm range. The chemical reduction of GO renders red shift of λ_max_ up to 270 nm depending on the degree of reduction due to the recovery of graphitic sp^2^ conjugation^18^. λ_max_ for p-rGO suggests that sp^2^ conjugation both in p-GO and p-rGO is almost identical. Vacuum filtration of p-rGO/PVP assembly solution provides p-rGO film without remaining P4VP because noncovalent interaction between p-rGO plate and P4VP chains is robust only in solution state^19^. FT-IR spectra of both p-rGO film and p-CF powder were almost identical as shown in Fig. 4(a), revealing complete disappearance of most C=O and C-O stretching peaks. XPS analysis of p-rGO film revealed that p-rGO has 88.1% of non-reduced carbon (including graphitic carbon) and 11.9% of oxidized carbon as shown in Fig. 5(c). Considering the fact that typical O/C ratio of rGO (from the reduction of GO with hydrazine) is 1:10^8^, portion of graphitic carbon is believed to stay in p-rGO. Therefore, it is regarded that chemical reduction of p-GO couldn’t increase domain size of conjugated sp^2^ carbons in p-rGO. The plate dimension of both p-GO and p-rGO seems to be unchanged after chemical reduction because DLS showed preservation of particle diameter at around 1 μm even after reduction to p-rGO as shown in Fig. S3. Meanwhile, weak fluorescence emission at 430 nm of p-GO significantly decreases below 5% of original fluorescence intensity after chemical reduction as shown in Fig. S4. This fluorescence intensity decrease of GO after chemical reduction is often observed in rGO due to the recovery of graphitic sp^2^ conjugation^20^. Therefore, recovery of sp^2^ conjugation in p-rGO is estimated to be comparable or weaker than rGO. Recently, carbon nanosheets (CNNs) derived from petroleum pitch have been recently reported and electrical conductivity of 30,000 S/m was demonstrated for 2 nm thick CNN film after carbonization of spin-coated pitch on silicon wafer^21^. 10 nm thick p-rGO thin film obtained by reduction of p-GO film by hydrazine vapor and thermal treatment at 400 °C demonstrates conductivity of 2,700 S/m with optical transmittance of 85% at 550 nm as shown in Fig. 8(b). More detailed research work is required to fully understand molecular structures and electronic transfer characteristics of both p-GO and p-rGO for the application of them for molecular electronics.

## Discussion

### p-GO-based Polymer Nanocomposites

**Figure 9:**
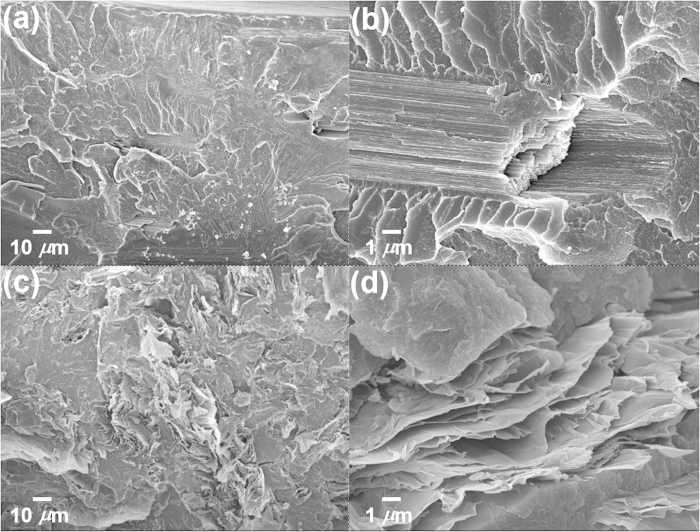
SEM images of (**a**) fractured section of p-CF/PMMA composite (2 wt.-% of p-CF), (**b**) its magnification (×10), (**c**) fractured section of p-GO/PMMA composite (2 wt.-% of p-GO), and (**d**) its magnification (×10).

Incorporating graphene-based materials into the polymer matrix can significantly improve mechanical properties of the polymer. Therefore, it is interesting to validate p-GO as nanofiller for polymer nanocomposite because of 2D structural characteristic and ultrathin (single atomic thick) nature of p-GO. To formulate p-GO-based polymer nanocomposite, p-GO powder was mixed with either PMMA or PS with different loading of p-GO (1, 2, 3, and 4 wt.-%). p-GO/polymer composite sheets were prepared by hot press of p-GO/polymer compound which is produced by using a twin screw extruder. The effects of p-GO loading on the tensile properties of both PMMA and PS were summarized as shown in Table 1. The representative strain-stress curves of p-GO/PMMA composite were shown in Fig. S6. The tensile strength of p-GO/PMMA sheet containing 1 wt.-% of p-GO is increased by up to 64 MPa (about 230% greater than that of neat PMMA). Increasing p-GO content from 1 to 3 wt.-% further increases the tensile strength to 67 MPa, which decreased to 53 MPa at 4 wt.-% loading of p-GO. Interestingly, the elongation at break of nanocomposite sheet was increased from 0.82% of neat PMMA to 3.06% at 3 wt.-% loading of p-GO and then decrease to 1.99% at 4 wt.-% loading of p-GO. The modulus of toughness is defined as the total area up to fracture in stress-strain curve of material and corresponds to the energy needed to completely fracture the material. Significant increase of both tensile strength and the elongation at break results in dramatic increase of the modulus of toughness of p-GO/PMMA composite. p-GO/PMMA nanocomposite having 3 wt.-% of p-GO loading showed 10.2 × 10^7^ N/m^2^ of the modulus of toughness (about 900% greater than that of neat PMMA). The fact that even p-GO/PMMA composite having 1.0 wt.-% loading shows 9.49 × 10^7^ N/m^2^ of the modulus (about 830 wt.-% greater than that of neat PMMA) clearly shows that both inclusion and dispersion of p-GO in PMMA matrix is quite effective. Until now, mechanical reinforcement of most graphene based polymer nanocomposites has shown increase of tensile strength together with decrease of the elongation at break, resulting in no significant change of the modulus of toughness of polymer nanocomposite. Therefore, it can be regarded that structural features of p-GO/PMMA nanocomposite is different with the other graphene-based polymer nanocomposites^22^. XRD analysis of p-GO/PMMA composite with 1 or 3 wt.-% loading of p-GO only showed broad amorphous scattering peak of neat PMMA in as shown in Fig. S5. Meanwhile p-GO didn’t show meaningful reinforcement for PS regardless of p-GO loading amount. In addition, p-CF itself didn’t show any reinforcement even on PMMA with 2 wt.-% of p-CF loading and p-GO also didn’t contribute to reinforcement of PS. The mismatch of surface energy between p-GO and PS might result in such weak reinforcement for PS in p-GO/PS nanocomposite. For example, surface-modification of p-GO with hydrophobic long alkyl chains might be helpful for the construction of mechanically reinforced PS nanocomposite with p-GO^12^,^23^. Therefore, it is concluded that 2D planar structural feature, single atomic thickness, and hydrophilic nature of p-GO contribute to significant reinforcement of polar PMMA. The extent of nanofiller dispersion in polymer matrix closely correlates with its effectiveness for improving mechanical, electrical, thermal, and other properties. SEM image of p-GO/PMMA composite having 2 wt.-% of p-GO as nanofiller showed composite structure with entirely filled with p-GO sheets as shown in Fig. 9. While most of p-GO sheets are crumpled, wrinkled, folded, the presence of effectively dispersed p-GO sheets compromise high surface area of p-GO sheets, which results in effective reinforcement of host PMMA. SEM image of p-CF/PMMA composite having 2 wt.-% of p-CF as nanofiller showed composite structure having embedded shortened fibers without change of diameter. While the mechanical reinforcement of polymer matrix by the inclusion of p-GO, GO-like 2D carbon nanomaterial, as nanofiller is clearly demonstrated, the reinforcement behavior of p-GO is very unique because the modulus of toughness rather than the modulus is increased in most reports^22^. This unique reinforcing behavior of p-GO might come from the structural feature of p-GO with slightly higher aspect ratio of p-GO. Slight modification of the morphological features (spherical, tubular, and plate) of nanofillers induces significant effects on the physical properties of polymer nanocomposites^24^. While p-GO is similar to GO, p-GO has slight 1D structural feature because the aspect ratio of p-GO is between 1.0 ~ 1.3, which inevitably comes from the 1D structural feature of pristine p-CF with high aspect ratio.

In conclusion, p-GO was successfully prepared by chemical exfoliation of p-CF. p-GO is regarded as alternative GO because most of compositional and structural features of both materials are similar. While electrical and thermal properties of p-rGO is expected to be less than either rGO or other exfoliated graphene nanosheets due to higher sp^3^ defect density of p-GO compared with GO, the success of p-GO for the mechanical reinforcement of PMMA nanocomposite is promising for the enlargement of the category of graphene-based nanomaterials to accomplish the formulation of high performance nanocomposite.

## Methods

### Materials and instruments

Chopped p-CF (ThermalGraph® DKD) was purchased from Cytec Industries and poly(4-vinylpyridine) (P4VP) (weight average molecular weight of 60,000 Da) was purchased from Sigma-Aldrich Corporation. Polystyrene (PS) and PMMA resins were provided from LG chemical Ind. All the other chemicals were purchased from Sigma-Aldrich Corporation and used without further purification. Ultraviolet-Visible (UV-Vis) spectra were obtained from UV-Vis spectrometer of Hewlett Packard. Photoluminescence (PL) spectra were obtained from a Perkin-Elmer Luminescence spectrometer L550B at room temperature (excited at 300 nm). Fourier-Transform Infrared (FT-IR) spectra were obtained from Nicolet IR 200 (USA). Dynamic light scattering (DLS) data were obtained from particle size analyser (ELS-Z) of Otsuka Electronics Corporation with temperature controller. Atomic force microscopy (AFM) images were obtained from XE-100 model of PSIA. Transmission electron microscopy (TEM) images were recorded with FEI Tecnai T20 at an accelerating voltage of 200 kV. Raman analysis was done from LabRAM high resolution UV/VIS/NIR dispersive Raman microscope of Horiba John Yvon. X-ray photoelectron spectroscopy (XPS) data were obtained from Multilab 2000 of Thermo Scientific, and the binding energies are with respect to graphitic carbon C1s at 284.5 eV. The binding energy is accurate to within ±0.1 eV. X-ray diffraction (XRD) data were obtained from D8 Advance X-ray Diffraction of Bruker Instrument with Cu Kα radiation (λ = 1.5405 Å).

### Production of p-GO

5.00 g of p-CF was mechanically stirred with 200 mL of H_2_SO_4_, 4.00 g of potassium persulfate (K_2_S_2_O_8_), and 4.00 g of phosphorus pentoxide (P_2_O_5_) at 80 °C for 5 hrs. After cooling to room temperature, reaction mixture was carefully added into 800 mL of deionized water and magnetically stirred at room temperature for 24 hrs. Dark brown solid was isolated by filtration and briefly washed with deionized water. Vacuum drying of above solid at room temperature for 24 hrs produced pre-oxidized p-CF. Then, pre-oxidized p-CF was mechanically stirred with 200 mL of H_2_SO_4_ at 0 °C and 25 g of potassium permanganate (KMnO_4_) was slowly added while keeping reaction temperature below 20 °C. Whole the reaction mixture was mechanically stirred at 35 °C for 2 h and 400 mL of water was slowly added into the mixture for 2 h while keeping reaction temperature below 50 °C. Finally, the reaction mixture was carefully added into 23.3 L of deionized water and 30 mL of hydrogen peroxide (H_2_O_2_) solution (20 wt.-% in water) was added dropwise. The crude p-GO powder was washed with both HCl solution (10 wt.-% in water) and deionized water until pH of solution is maintained. To obtain purified p-GO, crude p-GO was sonicated for 30 min in deionized water and any non-oxidized carbon was removed by centrifuge (5000 rpm, for 1 hr). Finally, filtration of p-GO solution was attempted through cotton fiber mesh, resulting in optically clear purified p-GO solution. Purified p-GO powder (7.50 g) was obtained by freeze drying of this solution.

Chemical reduction of p-GO to p-rGO. Fresh p-GO solution (1.0 mg of p-GO in 10 mL of deionized water) was prepared with minimum sonication (no more than 30 min below 10 °C) just before use. Then, 10 mg of P4VP in 25 mL of 10 wt.-% aqueous acetic acid was simply magnetically mixed with p-GO solution, resulting in bright brown p-GO/P4VP mixture solution. To chemically reduce p-GO into p-rGO, four drops of hydrazine monohydrate (30 wt.-% aqueous solution) were added into this p-GO/P4VP mixture solution and the reaction temperature was maintained at 80 °C for 24 hrs, producing dark black colored p-rGO/P4VP assembly solution with optical clarity. Prepared p-rGO/P4VP assembly was stable for more than 6 months without any visible precipitation. p-rGO film was obtained by filtration of above p-rGO/P4VP assembly solution on anodized aluminium oxide (AAO) membrane with pore size of 20 nm.

### p-GO/polymer nanocomposites

Inclusion of p-GO into polymer matrix was performed by using Rheocord 9000 twin screw extruder of Haake with 600 rpm at 180 °C for 10 min. p-GO/polymer composite sheets were obtained from Fuse MP-50 hot press of Fusei Menix at 180 °C for 10 min. Tensile tests of p-GO/polymer nanocomposite sheets (width of 12.75 mm and thickness of 3.25 mm) were obtained from Instron 5567 universal tensile tester (Instron) with cross head speed of 5 mm/s based on ASTM D638 (relative humidity of 50% at 25 °C).

## Additional Information

**How to cite this article**: Lee, M. *et al.* Production of graphene oxide from pitch-based carbon fiber. *Sci. Rep.* **5**, 11707; doi: 10.1038/srep11707 (2015).

## Supplementary Material

Supplementary Information

## Figures and Tables

**Figure 1:**
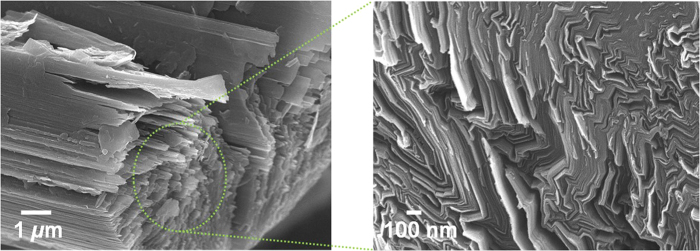
SEM images of weakly exfoliated p-CF (after immersing p-CF in H_2_SO_4_/HNO_3_ (3:1) for 32 hrs at r. t.).

**Figure 2:**
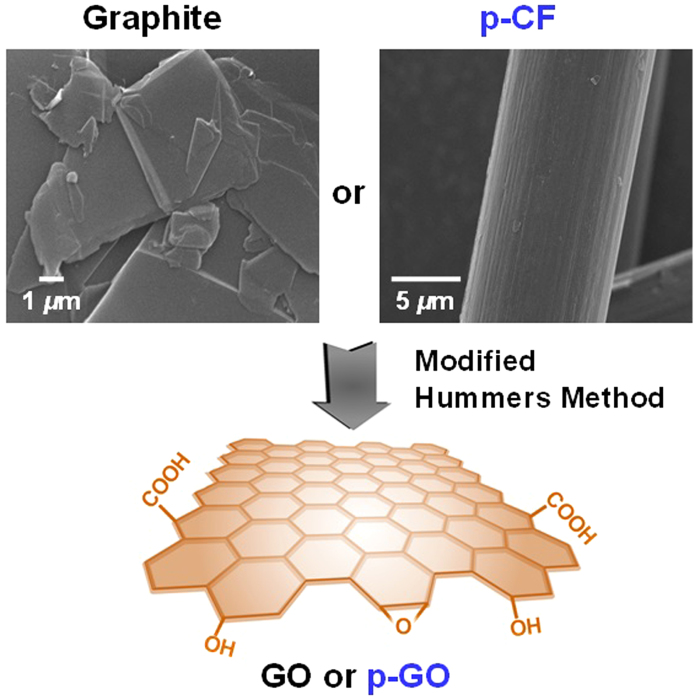
A representative scheme for the preparation of either GO or p-GO from either graphite or p-CF by modified Hummers method (SEM images of both graphite and p-CF are inserted).

**Table 1:** Tensile properties of polymer composites.

Composite	σ_b_^a^ (MPa)	ε_b_^b^ (%)	U^c^ (N/m^2^)
Neat PMMA	27.9 ± 2.3	0.82 ± 0.06	(1.1 ± 0.22)×10^7^
p-GO/PMMA (1 wt.-% p-GO)	63.9 ± 4.5	2.97 ± 0.19	(9.49 ± 0.8)×10^7^
p-GO/PMMA (2 wt.-% p-GO)	64.4 ± 3.9	2.78 ± 0.32	(8.93 ± 1.05)×10^7^
p-GO/PMMA (3 wt.-% p-GO)	66.8 ± 6.1	3.06 ± 0.23	(10.2 ± 0.85)×10^7^
p-GO/PMMA (4 wt.-% p-GO)	53.3 ± 4.3	1.99 ± 0.47	(5.29 ± 0.74)×10^7^
p-GO/PMMA (2 wt.-% CF)	28.5 ± 3.9	0.80 ± 0.09	(1.1 ± 0.06)×10^7^
Neat PS	28.7 ± 3.8	0.88 ± 0.16	(1.3 ± 0.12)×10^7^
p-GO/PS (2 wt.-% p-GO)	36.8 ± 2.1	0.93 ± 0.07	(1.7 ± 0.09)×10^7^

^a^tensile strength,

^b^elongation at break,

^c^modulus of toughness.

## References

[b1] Chen D., Feng H., Li J. Graphene oxide: preparation, functionalization, and electrochemical applications. Chem Rev. 112, 6027 (2012) 22889102 10.1021/cr300115g

[b2] Kim K. *et al.* Large-scale pattern growth of graphene films for stretchable transparent electrodes. Nature 457, 706 (2009) 19145232 10.1038/nature07719

[b3] Zhang C. *et al.* Towards low temperature thermal exfoliation of graphite oxide for graphene production. Carbon 62, 11 (2013)

[b4] Hernandez Y. *et al.* High-yield production of graphene by liquid-phase exfoliation of graphite. Nat Nanotechnol. 3, 563 (2008) 18772919 10.1038/nnano.2008.215

[b5] Georgakilas V. *et al.* Functionalization of Graphene: Covalent and Non-Covalent Approaches, Derivatives and Applications. Chem Rev. 112, 6156 (2012) 23009634 10.1021/cr3000412

[b6] Watanabe F. *et al.* Pitch-based carbon fiber of high compressive strength prepared from synthetic isotropic pitch containing mesophase spheres. Carbon 37, 961 (1999)

[b7] Peng J. *et al.* Graphene quantum dots derived from carbon fibers. Nano Lett. 12, 844 (2012) 22216895 10.1021/nl2038979

[b8] Marcano D. C. *et al.* Improved synthesis of graphene oxide. ACS Nano. 4 , 4806 (2010) 20731455 10.1021/nn1006368

[b9] Kudin K. N. *et al.* Raman spectra of graphite oxide and functionalized graphene sheets. Nano Lett. 8, 36 (2008) 18154315 10.1021/nl071822y

[b10] Cançado L. G. *et al.* Measuring the degree of stacking order in graphite by Raman spectroscopy. Carbon 46, 272 (2008)

[b11] Hontoria-Lucas C. *et al.* Study of oxygen-containing groups in a series of graphite oxides: physical and chemical characterization. Carbon 33, 1585 (1995)

[b12] Stankovich S. *et al.* Graphene-based composite materials. Nature 442, 282 (2006) 16855586 10.1038/nature04969

[b13] Dreyer D. R., Park S., Bielawski C. W., Ruoff R. S. The chemistry of graphene oxide. Chem Soc Rev. 39, 228 (2010) 20023850 10.1039/b917103g

[b14] Lee D. Y. *et al.* Blood compatible graphene/heparin conjugate through noncovalent chemistry. Biomacromolecules 12, 336 (2011) 21218769 10.1021/bm101031a

[b15] Lee D. Y. *et al.* Thermo-Responsive Assembly of Chemically Reduced Graphene and Poly(*N*-isopropylacrylamide). Macromol Chem Phys. 212, 336 (2011)

[b16] Ha S. G. *et al.* Formation of Semiconducting Chemically Reduced Graphene Oxide/Cellulose Assembly through Noncovalent Interactions Chem Lett. 42, 1409 (2013)

[b17] Lee M. Y. *et al.* Formulation of chemically reduced graphene oxide assembly with poly(4-vinyl pyridine) through noncovalent interaction. J Appl Polym Sci. 130, 2538 (2013)

[b18] Wang Y., Shi Z. & Yin J. Facile synthesis of soluble graphene via a green reduction of graphene oxide in tea solution and its biocomposites. ACS Appl Mater Inter. 3, 1127 (2011) 10.1021/am1012613 21438576

[b19] Chua C. K. & Pumera M. Chemical reduction of graphene oxide: a synthetic chemistry viewpoint. Chem Soc Rev. 43, 291 (2014) 24121318 10.1039/c3cs60303b

[b20] Loh K. P., Bao Q., Eda G. & Chhowalla M. Graphene oxide as a chemically tunable platform for optical applications. Nat Chem. 2, 1015 (2010) 21107364 10.1038/nchem.907

[b21] Lee J. S., Joh H. I., Kim T. W. & Lee S. Carbon nanosheets derived from soluble pitch molecules and their applications in organic transistors. Org Electron. 15, 132 (2014)

[b22] Kim H., Abdala A. A. & Macosko C. W. Graphene/polymer nanocomposites. Macromolecules 43, 6515 (2010)

[b23] Potts J. R., Dreyer D. R., Bielawski C. W. & Ruoff R. S. Graphene-based polymer nanocomposites. Polymer 52, 5 (2011)

[b24] Reddy C. S., Zak A. & Zussman E. WS_2_ nanotubes embedded in PMMA nanofibers as energy absorptive material. J. Mater. Chem. 21, 16086 (2011).

