# Supplementary material for: Production of graphene oxide from pitch-based carbon fiber
Source: Sci Rep. 2015 Jul 9;5:11707. doi: 10.1038/srep11707 (PMC4648413; doi:10.1038/srep11707)
Supplement: Supplementary Information [file srep11707-s1.pdf]

Supplementary Information

Production of graphene oxide from pitch-based carbon fiber

Miyeon Lee,[1] Sungyoung Park,[2,3] Jihoon Lee,[3,4] Byunggak Min,*[4] Bongsoo Kim,*[1] and Insik In*[3,4]

[1]Department of Chemistry, KAIST, Daejeon, 305-701, South Korea, [2]50 Daehak-ro, Department of Chemical and Biological Engineering, Korea National University of transportation, Chungju-si, Chungbuk, 380-702, South Korea, [3]50 Daehak-ro, Department of IT Convergence (BK PLUS 21), Korea National University of Transportation, Chungju-si, Chungbuk, 380-702, South Korea, [4]50 Daehak-ro, Department of Polymer Science and Engineering, Korea National University of Transportation, Chungju-si, Chungbuk, 380-702, South Korea.

* Correspondence and requests for materials should be addressed I. I. (in1@ut.ac.kr)

Supplementary Figures

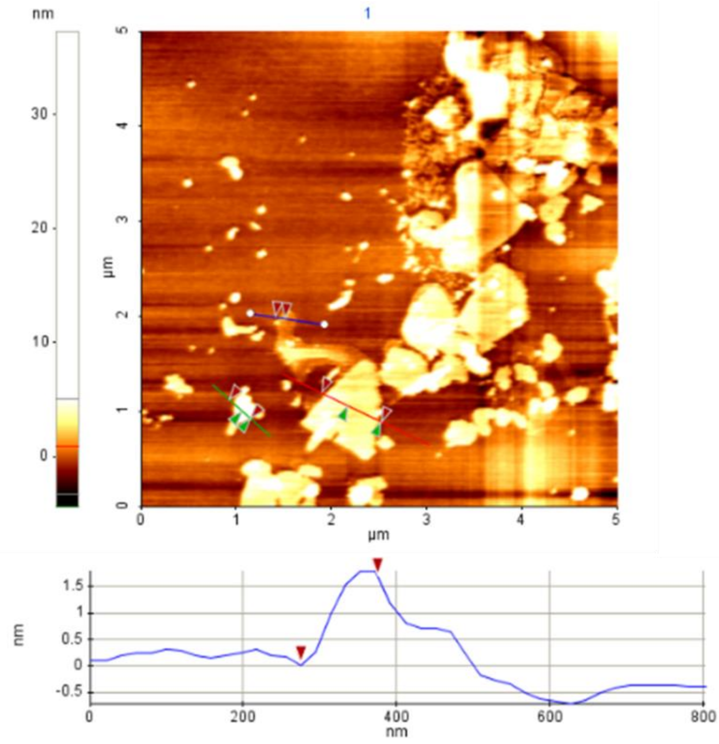

Fig. S1 AFM image of p-GO deposited on silicon wafer (bottom is line profile for the red line in image).

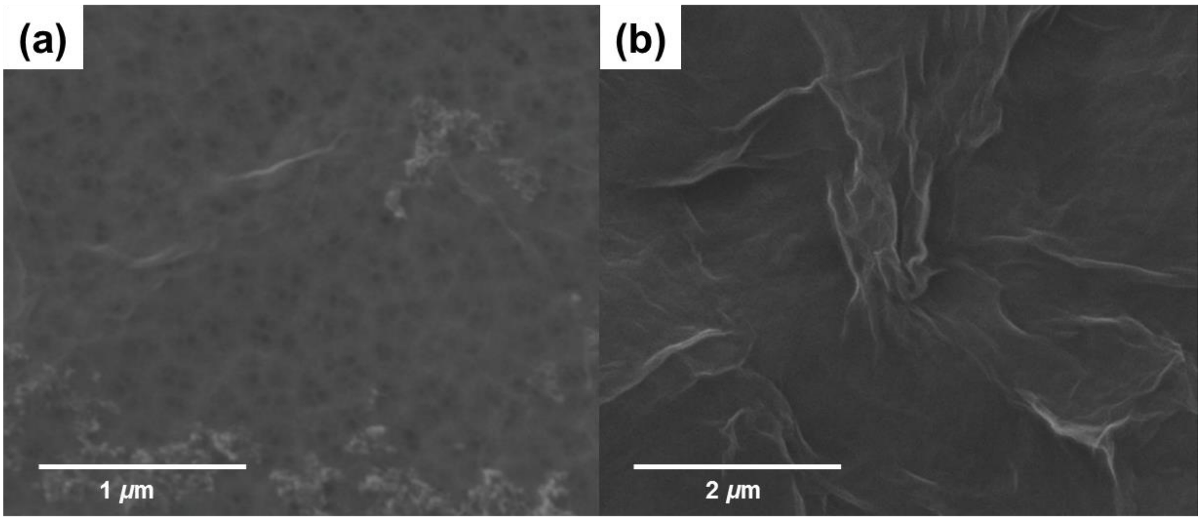

Fig. S2 SEM images of (a) thin p-GO film and (b) thick p-GO film on anodized aluminum oxide (AAO) membrane (underlying structure is pores of AAO membrane below p-GO film).

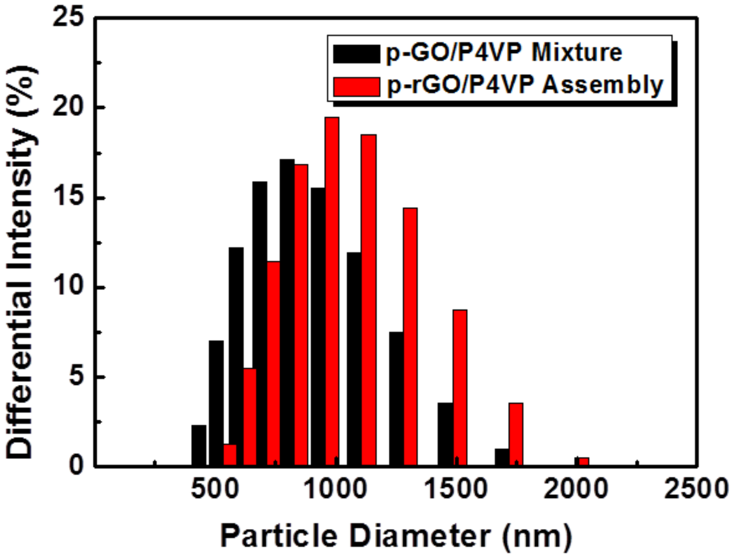

Fig. S3 Particle diameter distributions of p-GO/P4VP mixture (1:10) and p-rGO/P4VP assembly solutions.

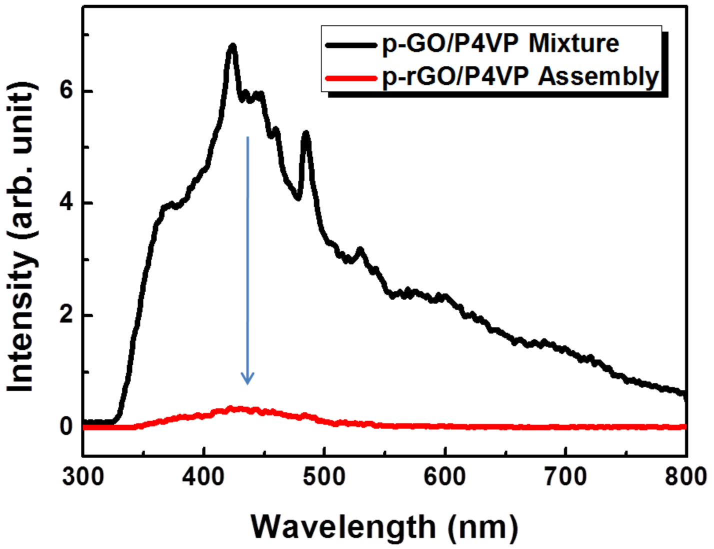

Fig. S4 PL spectra of p-GO/P4VP (1:10) mixture and p-rGO/P4VP assembly solutions (excitation wavelength of 300 nm).

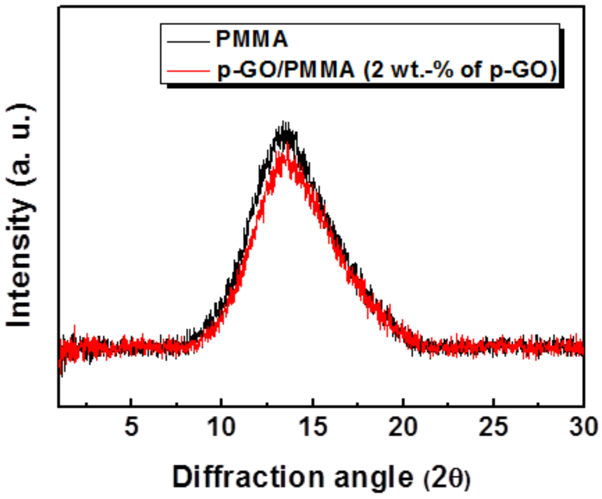

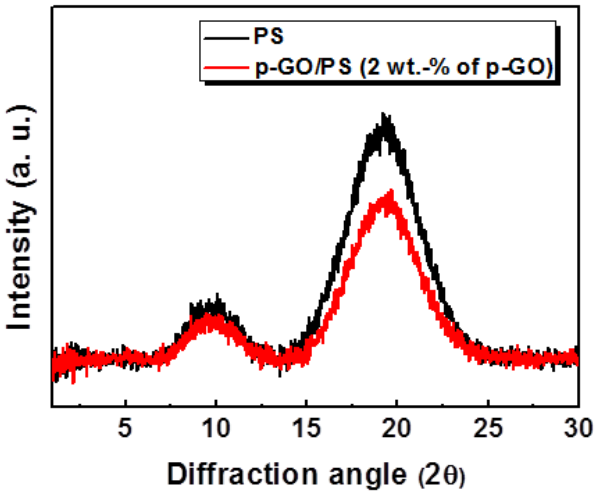

Fig. S5 XRD data of polymers and their composites with either p-GO or p-CF.

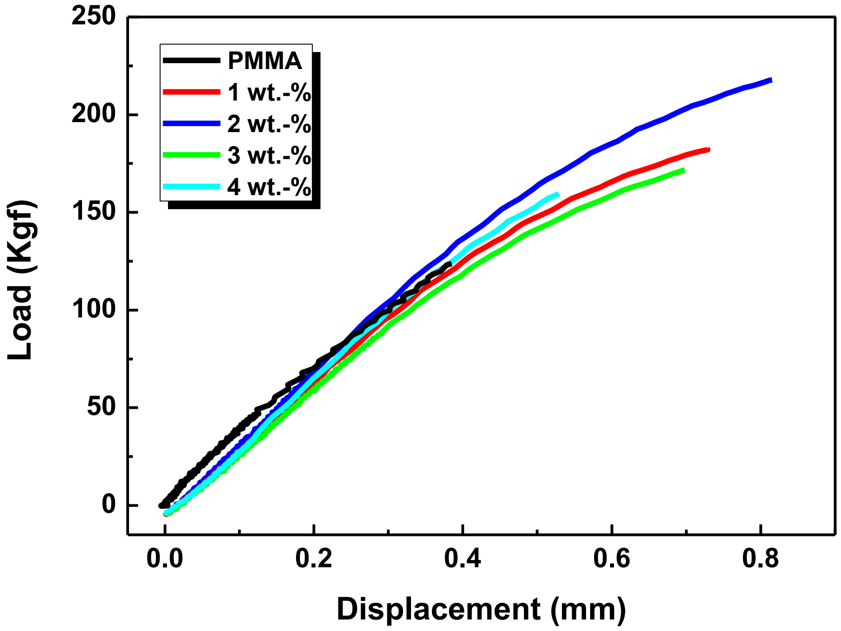

Fig. S6 Representative stress-strain curves for both PMMA and p-GO/PMMA composites depending of the amount of p-GO in the composite.
